# The role of textiles as fomites in the healthcare environment: a review of the infection control risk

**DOI:** 10.7717/peerj.9790

**Published:** 2020-08-25

**Authors:** Lucy Owen, Katie Laird

**Affiliations:** Infectious Disease Research Group, The Leicester School of Pharmacy, De Montfort University, Leicester, United Kingdom

**Keywords:** Textiles, Linen, Healthcare uniforms, Fomite, Laundry, Laundering, Decontamination, Infection control

## Abstract

**Background:**

Infectious diseases are a significant threat in both healthcare and community settings. Healthcare associated infections (HCAIs) in particular are a leading cause of complications during hospitalisation. Contamination of the healthcare environment is recognised as a source of infectious disease yet the significance of porous surfaces including healthcare textiles as fomites is not well understood. It is currently assumed there is little infection risk from textiles due to a lack of direct epidemiological evidence. Decontamination of healthcare textiles is achieved with heat and/or detergents by commercial or in-house laundering with the exception of healthcare worker uniforms which are laundered domestically in some countries. The emergence of the COVID-19 pandemic has increased the need for rigorous infection control including effective decontamination of potential fomites in the healthcare environment. This article aims to review the evidence for the role of textiles in the transmission of infection, outline current procedures for laundering healthcare textiles and review studies evaluating the decontamination efficacy of domestic and industrial laundering.

**Methodology:**

Pubmed, Google Scholar and Web of Science were searched for publications pertaining to the survival and transmission of microorganisms on textiles with a particular focus on the healthcare environment.

**Results:**

A number of studies indicate that microorganisms survive on textiles for extended periods of time and can transfer on to skin and other surfaces suggesting it is biologically plausible that HCAIs and other infectious diseases can be transmitted directly through contact with contaminated textiles. Accordingly, there are a number of case studies that link small outbreaks with inadequate laundering or infection control processes surrounding healthcare laundry. Studies have also demonstrated the survival of potential pathogens during laundering of healthcare textiles, which may increase the risk of infection supporting the data published on specific outbreak case studies.

**Conclusions:**

There are no large-scale epidemiological studies demonstrating a direct link between HCAIs and contaminated textiles yet evidence of outbreaks from published case studies should not be disregarded. Adequate microbial decontamination of linen and infection control procedures during laundering are required to minimise the risk of infection from healthcare textiles. Domestic laundering of healthcare worker uniforms is a particular concern due to the lack of control and monitoring of decontamination, offering a route for potential pathogens to enter the clinical environment. Industrial laundering of healthcare worker uniforms provides greater assurances of adequate decontamination compared to domestic laundering, due to the ability to monitor laundering parameters; this is of particular importance during the COVID-19 pandemic to minimise any risk of SARS-CoV-2 transmission.

## Introduction

Infectious diseases are a leading cause of morbidity and mortality in the community and healthcare settings worldwide (GBD 2017 Disease and Injury Incidence and Prevalence Collaborators, 2018; GBD 2017 Causes of Death Collaborators, 2018). The public health threat associated with infectious diseases has been further highlighted in recent years with the rise in antimicrobial resistance (Logan & Weinstein, 2017) and onset of the Coronavirus Disease 2019 (COVID-19) pandemic (Jin et al., 2020). Infection control interventions are important to reduce the spread of infectious disease; hand hygiene and disinfection of surfaces are considered key infection control measures (Otter et al., 2016) yet less emphasis has been placed on the disinfection of soft surfaces, suggesting that the transmission of infection by textiles could be potentially overlooked (Fijan & Turk, 2012; Mitchell et al., 2015).

Healthcare associated infections (HCAIs) are one of the most frequent complications of hospitalisation and a significant threat to patient safety. HCAIs lead to increased hospital stays, morbidity, mortality and treatment costs (Allegranzi et al., 2013). The prevalence of HCAIs is estimated to be 7.6% in developed countries and 5.7–19.1% in developing countries (World Health Organization, 2011). HCAIs arise primarily through the patients’ endogenous flora, yet contamination of the healthcare environment plays a significant role in the transmission of exogenous HCAIs (Weber et al., 2010). Potential pathogens such as methicillin resistant *Staphylococcus aureus* (MRSA), vancomycin resistant *Enterococcus* sp. (VRE)*, Clostridioides difficile* spores and norovirus are shed by infected patients and/or healthcare workers and repeatedly contaminate the surrounding clinical environment. Such pathogens can survive on surfaces for months, posing the risk of transmission to patients by direct or indirect contact (Weber et al., 2010; Otter, Yezli & French, 2014; Dancer & Kramer, 2019). In accordance, a significant risk factor for the acquisition of HCAI is admission to a hospital room previously occupied by a carrier of the same HCAI (Mitchell et al., 2015) and decontamination of rooms occupied by *C. difficile* patients have been shown to reduce the rate of subsequent infections (Donskey, 2013). Cleaning and disinfection is considered an important infection control strategy to reduce surface contamination and prevent HCAIs (Dancer & Kramer, 2019).

The emergence of Severe Acute Respiratory Syndrome Coronavirus 2 (SARS-CoV-2) and the subsequent COVID-19 pandemic has led to a further need for rigorous infection control interventions, with person-to-person transmission of SARS-CoV-2 being observed in both healthcare and community settings (Chan et al., 2020; Heinzerling et al., 2020). SARS-CoV-2 is predominantly transmitted through respiratory droplets in addition to contact with contaminated objects (World Health Organization, 2020), with some evidence to suggest the involvement of aerosols in transmission (Prather, Wang & Schooley, 2020). Contamination of the healthcare environment may increase the risk of nosocomial COVID-19 transmission (Otter et al., 2016; Kampf et al., 2020).

Studies investigating the role of inanimate objects in the transmission of HCAIs and infection control strategies have primarily focused on medical instruments and high-touch non-porous objects e.g., bed rails and door handles (Haun, Hooper-Lane & Safdar, 2016; Mody et al., 2019; Smith et al., 2016; Facciola et al., 2019). There is a paucity of studies investigating the role of soft surfaces and healthcare textiles in the transfer of microorganisms or HCAI acquisition, despite work demonstrating that they readily become contaminated with microorganisms (Heudorf et al., 2017). It is currently assumed that the risk of infection from textiles is low, and there is less emphasis on the decontamination of textiles compared to non-porous surfaces (Loveday et al., 2007, Fijan & Turk, 2012; Mitchell et al., 2015). Decontamination of healthcare linens is achieved by laundering in industrial or in-house facilities using high temperatures (≥60 °C) and detergents (Bockmühl, Schages & Rehberg, 2019), whereas healthcare worker uniforms are laundered domestically in the UK, Republic of Ireland and in some hospitals in the US (Nordstrom, Reynolds & Gerba, 2012; NHS, 2020), meaning that it is not possible to monitor decontamination. Recent studies have demonstrated the survival of potential pathogens on textiles during both domestic and industrial laundering (Heudorf et al., 2017; Riley et al., 2017; Tarrant, Jenkins & Laird, 2018), indicating that further studies are warranted to investigate the infection risk associated with contaminated linen. This is of particular importance during the COVID-19 pandemic.

This article aims to review the current literature surrounding the role of healthcare textiles in the transmission of HCAIs, outline current procedures for laundering healthcare textiles and review studies evaluating their decontamination efficacy.

## Survey Methodology

Pubmed, Google Scholar and Web of Science were searched for articles relating to the survival of microorganisms on textiles, contamination of healthcare textiles and the transmission of HCAIs published on or before May 2020. Further references were obtained from studies mentioned in literature reviews, systematic reviews and meta-analyses. Selection of relevant articles was not performed systematically but was fully objective and used to compare and contrast current evidence and hypotheses.

### Contamination of healthcare textiles

Microorganisms are shed from infected and colonised patients or staff into the environment (Otter, Yezli & French, 2014). In particular, reusable healthcare textiles in close contact with patients for extended periods of time can become soiled with bodily fluids, blood and skin scales, leading to contamination with potential pathogens (Creamer & Humphreys, 2008). Pathogens linked to HCAIs have been shown to contaminate textiles in the clinical environment; 51 CFU/25 cm^2^ *C. difficile* spores were recover from soiled hospital bed sheets of *C. difficile* infection patients (Tarrant, Jenkins & Laird, 2018) and 92% of privacy curtains are contaminated with at least one bacterial species within one week, including *S. aureus* (62% positive), MRSA (21% positive) and VRE (42% positive) (Ohl et al., 2012).

In addition to the near-patient environment becoming contaminated, healthcare worker attire can also become contaminated during contact with patients, with approximately 10% of healthcare worker gowns becoming contaminated with microorganisms from patients during simulated healthcare activities (Wolfensberger et al., 2018). The rate of transfer of microorganisms to hospital gowns (10%) was lower than hands and gloves (30–33%) (Wolfensberger et al., 2018). Personal protective equipment (PPE) such as gowns and plastic aprons protect healthcare worker uniforms from exposure to bodily fluids during clinical activities, in turn protecting from contamination with potential pathogens. Self-contamination with microorganisms may occur during the removal of PPE; Casanova et al. (2008) reported that 1–4 log_10_ MS2 bacteriophage transferred onto sterile scrubs during removal of artificially contaminated PPE (gloves and gowns) by 10 healthcare workers following simulated care activities, indicating that healthcare worker uniforms are at risk of contamination with potential pathogens.

In accordance, healthcare worker uniforms were found to become increasingly contaminated with microorganisms during wear; MRSA, VRE and/or *C. difficile* were present on 39% of nurse’s uniforms (1 to >100 colony forming units (CFU), before their shift, increasing to 54% at the end of the shift (Perry, Marshall & Jones, 2001). Similarly, Burden et al. (2011) demonstrated that freshly laundered doctors’ scrub uniforms became increasingly contaminated over an 8-hour shift, within 2.5 h the pockets alone were contaminated with around 50 CFU total viable count, increasing to >100 CFU after 8 h and MRSA was present on 20% of the uniforms sampled (Burden et al., 2011).

There has been debate on the safety of doctors’ traditional work attire of white coats and neckties in relation to the transfer of microorganisms, because they are rarely laundered compared to scrub uniforms (Weber et al., 2012). *S. aureus,* MRSA and Gram-negative rods were identified on 3–79% of doctors’ white coats and 8–52% of neckties, while white coats had a greater percentage of the potential pathogens *Acinetobacter* spp., *S. aureus* and/or *Enterococcus* spp. (45.4%) compared to scrub uniforms (28.8%) (Haun, Hooper-Lane & Safdar, 2016). Although the differences were not significant (*p* > 0.05) (Munoz-Price et al., 2012; Goyal et al., 2019), the higher rate of contamination of white coats may be attributed to less frequent laundering; one survey concluded that white coats were washed on average every 12.4 days compared to every 1.7 days for scrubs (Munoz-Price et al., 2013; Goyal et al., 2019). Conversely, Burden et al. (2011) identified no significant differences in the total colony counts between white coats, which were rarely laundered, and freshly laundered scrub uniforms (104 versus 142 CFU). A systematic review reported that there was limited evidence that neckties were contaminated with pathogenic microorganisms. Only one randomised controlled trial was identified in the published literature, which reported that there were no pathogenic microorganisms isolated from neckties. The remaining studies surveyed were case-control and case series studies, which reported that neckties were contaminated with non-pathogenic environmental bacteria but provided little evidence of greater contamination with pathogens than not wearing a necktie (Pace–Asciak et al., 2018).

A disadvantage of current studies into the contamination of healthcare textiles is that many do not report the microbiological load or differentiate endogenous and environmental microorganisms which influences their risk as potential fomites; around one-third of microorganisms isolated from textiles are from the participants’ skin flora rather than healthcare-associated pathogens (Wilson et al., 2007). The majority of studies in the published literature do not attempt to correlate the observed microbiological contamination with the rate of HCAIs and thus do not provide evidence for contaminated textiles acting as fomites.

Microorganisms generally exhibit lower survival on porous surfaces than on non-porous surfaces (Bloomfield et al., 2015) yet they can survive on healthcare textiles for days to weeks (Table 1). *S. aureus*, *E. coli* and *E. faecium* survive on cotton for 21 days (Riley et al., 2017; Fijan, Pahor & Šostar Turk, 2017) and *S. aureus* and *E. faecium* survive on polyester for up to 7 days (Riley et al., 2017). Faecal coliforms also survive for 120 days on cotton and blended textile at 25 °C (>1.1 ×10^4^ CFU/ml), while few coliforms (1.1 ×10^2^ CFU/ml) survive on silk (Colclasure et al., 2015). The greater survival of microorganisms on cotton compared to polyester and silk can be partly attributed to the moisture content of the different fibres (Colclasure et al., 2015; Riley et al., 2017). Cotton absorbs moisture to a greater extent than synthetic materials such as polyester, which supports the enhanced survival of microorganisms on this fibre type (Riley et al., 2017). Spore forming bacteria exhibit even greater survival in the environment due to their resistance to desiccation, disinfection and high temperatures (Dyer et al., 2019); *C. difficile* spores have been reported to persist on dry surfaces for 5 months (Kramer, Schwebke & Kampf, 2006).

**Table 1:** In vitro survival of microorganisms on textiles.

**Microorganism**	**Surface**	**Survival**	**Reference**
*E. coli,* *S. aureus*	Cotton and polyester	5 log_10_ survived on cotton for 21 days; 0.16–0.28 log _10_ survived on polyester for 21 days	Riley et al. (2017)
*E. faecium, S. aureus, P. aeruginosa*	Cotton	4–5 log_10_*E. faecium* and *S. aureus* survived for 21 days. *P. aeruginosa* survived for 20 days.	Fijan, Pahor & Turk (2017)
Faecal coliforms	Cotton, blended textile and silk	Faecal coliforms survived for 120 days on cotton and blended textile at 25 °C (>1.1 ×10^4^ CFU/ml). 1.1 ×10^2^ CFU/ml survive on silk over 120 days.	Colclasure et al. (2015)
*Candida* spp., *Aspergillus* spp., *Fusarium* sp., *Mucor* sp., *Paecilomyces* sp.	Cotton, terry, blended textile, polyester and spandex	*Candida* spp. and *Aspergillus* spp. survived for 1 to >30. days *Fusarium* sp. for 4 to >30 days, *Mucor* sp. for 6 to >30 days and *Paecilomyces* sp. for <1 to 11 days.	Neely & Orloff (2001)
SARS-CoV	Cotton and disposable gowns	SARS-CoV survived on a cotton gown for 5 min at an inoculum of 10^4^ TCID_50_/ml and 24 h at an inoculum of 10^6^ TCID_50_/ml. Survival on a disposable gown was 1 h at 10^4^ TCID_50_/ml and 2 days at 10^6^ TCID_50_/ml.	Lai, Cheng & Lim (2005)
SARS-CoV-2	Cloth and surgical masks	SARS-CoV-2 persisted on cloth for 2 days, compared to 4 days on glass and bank notes to 7 days on surgical masks, stainless steel and plastic.	Chin et al. (2020)
HSV-1	Cotton	Herpes simplex virus 1 (HSV-1) in the presence of artificial soiling (bovine serum albumin and sheep erythrocytes) gradually reduces on cotton surfaces over time with a 1 log_10_ reduction after 30 min and complete inactivation within 48 h.	Gerhardts et al. (2016)
Poliovirus, adenovirus, hepatitis A virus and murine norovirus	Cotton, wool, gauze and diaper material	Poliovirus survives at room temperature for 84–140 days on wool and 42–84 days on cotton, adenovirus and hepatitis A remaining infectious for 60 days in cotton and murine norovirus surviving for 40 days on gauze and diaper material.	Yeargin et al. (2016)
HCoV OC43 and 229E	Cotton gauze sponge	HCoV 229E remained infectious for 12 h and OC43 for 3 h (initial titre 5 ×10^5^ TCID_50_/ml).	Sizun, Yu & Talbot (2000)

Fungal pathogens also survive on various hospital textiles (Table 1); *Candida* spp., *Aspergillus* spp., *Fusarium* sp., *Mucor* sp. and *Paecilomyces* sp. survived from one to >30 days on cotton, terry, blended textile, polyester and spandex (Neely & Orloff, 2001). The survival of viruses on textiles vary significantly depending on the species (Table 1). SARS-CoV remained infectious for 5 min to 24 h on cotton, depending on the initial titre, and for 48 h on disposable gowns (Lai, Cheng & Lim, 2005). SARS-CoV-2 persisted on cloth (unspecified material type) for 2 days, compared to 4 days on glass and bank notes to 7 days on surgical masks, stainless steel and plastic Chin et al. (2020). The human coronavirus (HCoV) OC43 was inactivated within 3 h on cotton gauze sponge, while HCoV 229E remained infectious for 12 h (Sizun, Yu & Talbot (2000). Herpes simplex virus 1 (HSV-1) in the presence of artificial soiling (bovine serum albumin and sheep erythrocytes) gradually reduces on cotton surfaces over time with complete inactivation within 48 h (Gerhardts et al., 2016). Enteric viruses survive for longer than SARS-CoV and HSV-1 on textiles, for example, poliovirus survives at room temperature for 84–140 days on wool and 42–84 days on cotton (Yeargin et al., 2016). The prolonged survival of enteric viruses compared to SARS-CoV and HSV-1 could be attributed to their lack of a lipid envelope; enveloped viruses are generally more susceptible to desiccation and other environmental conditions (Lucas, 2010).

In vitro studies of microbiological survival on textiles may not adequately reflect in use conditions. In particular, the loads of microorganisms employed are often high (such as 10^5^–10^9^ CFU/ml; Fijan, Šostar Turk Pahor & Šostar Turk, 2017; Riley et al., 2017) which increases survival duration (Fijan, Pahor & Šostar Turk, 2017). Natural contamination levels of healthcare textiles are likely to be smaller, for example 50 CFU was found on the sleeve cuff of doctors’ white coats (Burden et al., 2011) and 51 CFU/25 cm^2^ *C. difficile* spores were recovered from bed linen (Tarrant, Jenkins & Laird, 2018), indicating that the survival rates in vivo could be lower.

In vitro studies often use purified cultures of microorganisms without organic soiling which may not reflect conditions where microorganisms are present within organic matter such as bodily fluids (Creamer & Humphreys, 2008). The presence of soiling may affect the survival of some microorganisms on textiles, for example Abad, Pinto & Bosch (1994) reported that the survival of hepatitis A virus on cotton was lower in the presence of saline compared to 20% faecal suspension (1.6 versus 0.8 log_10_ reduction) whereas the survival of poliovirus on cotton was greater with saline compared to faecal suspension (2.7 versus 3.5 log_10_ reduction).

In addition, in vitro studies most commonly inoculate fabrics by pipetting microbiological suspensions on to the textile (Neely & Orloff, 2001; Lai, Cheng & Lim, 2005; Colclasure et al., 2015; Gerhardts et al., 2016; Riley et al., 2017; Chin et al., 2020). The use of microbiological suspensions simulates wet transfer of microorganisms, such as through respiratory fluids, whereas some pathogens may be transmitted without fluids, for example from a dry surface. This could lead to a difference in the survival of the microorganism on the textile which should be investigated further.

Despite the highlighted disadvantages of in vitro studies, the demonstrated persistence of microorganisms on textiles for several days indicates that textiles could potentially to act as a reservoir for the transmission of microorganisms, if the microorganisms are able to transfer to other surfaces in sufficient numbers to cause disease.

### The role of healthcare textiles in the transmission of infection

Microorganisms transmit from contaminated textiles through either direct or indirect contact, such as through contamination of healthcare workers’ hands or environmental surfaces and medical instruments. Healthcare worker uniforms could pose an enhanced risk of cross-contamination as the healthcare worker moves between patients (Mitchell et al., 2015). There is some in vitro evidence that microorganisms can be transferred from contaminated textiles to skin and objects (Table 2), however the majority of in vitro studies do not include any form of soiling which may not be realistic due to microorganisms transferring to textiles in blood, bodily fluids, skin scales etc. (Loveday et al., 2007; Creamer & Humphreys, 2008). MRSA has been shown to be transmissible from cotton bedsheets and towels onto porcine skin for up to 14 days (Desai et al., 2011); Table 2). In accordance, MRSA, VRE and pan drug resistant *Acinetobacter baumannii* transferred from 100% cotton white coats to porcine skin (Butler et al., 2010). The transfer of *E. coli, S. aureus, Bacillus thuringenesis* from cotton or polyester to fingertips was reportedly less efficient than non-porous surfaces (Lopez et al., 2013). Microorganisms may become embedded in the matrix of porous surfaces, such as the weave of textiles, leading to lower efficiency of transfer than non-porous surfaces, which may reduce their capacity to behave as fomites (Lopez et al., 2013). The transfer of microorganisms from environmental surfaces to textiles and *vice versa* has also been demonstrated by Dyer et al. (2019) who demonstrate that *C. difficile* spores were transferred from dry stainless steel and vinyl flooring to polypropylene laminate hospital gowns in vitro and were capable of transferring from contaminated to sterile hospital gowns (Table 2).

**Table 2:** In vitro studies on the transmission of microorganisms to/from textiles from other surfaces.

**Microorganism**	**Transfer material**	**Findings**	**Reference**
*Acinetobacter baumannii,* MRSA, VRE	100% cotton white coats to porcine skin	Test species transferred onto porcine skin 1, 5 and 30 min after textile inoculation with 0.5 MacFarland standard or a 1:100 dilution of this suspension. The rate of transfer was not quantified.	Butler et al. (2010)
*Bacillus thuringenesis,* *E. coli, S. aureus*	Cotton or polyester to fingertips	Transfer efficiencies of cotton and polycotton were <6.8–0.37% for *E. coli,* <1.0–0.37% for *S. aureus* and <0.6% for *B. thurengenesis.* Transfer was higher for non-porous surfaces at 40.7–3.8%, 20.3–2.7% and 57–0.04%, respectively.	Lopez et al. (2013)
*C. difficile* spores	Stainless steel or vinyl flooring to polypropylene laminate surgical gowns	10^1^-10^3^ CFU *C. difficile* spores transferred onto surgical gowns after 10 s to 1 min contact with stainless steel or vinyl surfaces spiked with 10^5^ CFU spores.	Dyer et al. (2019)
MRSA	Cotton bedsheets and towels to porcine skin	MRSA was transmissible for up to 14 days; 10^3^–10^4^ CFU transferred on to porcine skin 1 day after the textile was inoculated (10^6^ CFU inoculum) and 10^2^–10^3^ CFU transferred 7 days post-inoculation.	Desai et al. (2011)
*Acinetobacter calcoaceticus, E. coli* and *S. aureus*	Textile to textile: cotton, polycotton, polyester, silk, wool, polypropylene and viscose	Friction increased the transfer of *S. aureus* by two to five-fold and *E. coli* and *Acinetobacter calcoaceticus* by 5.7–61% compared to direct contact without friction. Transfer of *S. aureus, A. calcoaceticus* and *E. coli* was also significantly greater for wet fabrics compared to dry fabrics. *A. calcoaceticus* and *E. coli* transferred more efficiently from smoother textiles (viscose and polyester) compared to rougher textiles (polypropylene).	Varshney et al. (2020)
*S. aureus*	Textile (cotton/polycotton) to textile or fingers.	Transfer of *S. aureus* to fingers was generally low (<3% transfer), however polycotton had a greater rate of transfer than cotton. Friction increased transfer by up to 5-fold. Transfer was significantly greater from textile to other textile or fingers when the textile was moist and when friction was applied.	Sattar et al. (2001)

Key factors in the transfer of microorganisms from textiles include surface properties, friction and moisture of the fabric (Table 2). Friction increased the transfer of *S. aureus* by two to five-fold and *E. coli* and *Acinetobacter calcoaceticus* by 5.7–61% compared to direct contact without friction (Sattar et al., 2001; Varshney et al., 2020). Transfer of *S. aureus, A. calcoaceticus* and *E. coli* was also significantly greater for wet fabrics compared to dry fabrics (Sattar et al., 2001; Varshney et al., 2020). Surface roughness was concluded to decrease the transfer of microorganisms from textiles, due to greater transfer of *A. calcoaceticus* and *E. coli* from viscose and polyester compared to rougher polypropylene textiles (Varshney et al., 2020).

There are limited studies in the published literature that investigate the transfer of microorganisms to or from textiles in simulated or real-life clinical settings. The transfer of cauliflower mosaic virus DNA from mannequins to doctors’ uniforms during simulated physical examinations demonstrated that there was significantly greater contamination of long-sleeved white coats (approximately 25%) than short-sleeved uniforms (0%). This was likely the result of the sleeve cuffs touching the mannequin, which occurred during 44% of the observed interactions. Transmission of the cauliflower mosaic virus DNA to a second mannequin was limited and there was no significant difference between short and long-sleeved uniforms (*p* >  0.05; 5% versus 0%) (John et al., 2017). The rate of *Micrococcus luteus* transmission to mannequins during simulated physical examination was also statistically similar between short and long sleeve uniforms (*p* > 0.05; zero versus one of five mannequins contaminated) (Weber et al., 2012). Wearing a tie significantly (*p* ≤ 0.05) increased the contamination of mannequins with *M. luteus*; four out of five mannequins were contaminated following examination by a doctor wearing a long-sleeved uniform with a tie compared to one out of five without a tie (Weber et al., 2012). The studies by Weber et al. (2012) and John et al. (2017) suggest that there is little evidence for microorganisms transferring between patients from contaminated doctors’ uniforms with the exception of neckties. The studies are limited by the risk of doctors behaving differently upon being observed and the results may not reflect their usual practice. The study by John et al. (2017) is also limited by the use of viral DNA rather than a viable pathogen, which may have differing transfer efficiencies between textiles and the environment. There do not appear to be any similar investigations in the published literature on transmission from other healthcare textiles, such as bed linens, which are in more intimate contact with patients for longer periods of time. High-quality controlled trials are required to provide evidence for the transmission of potential pathogens, or lack thereof, from healthcare textiles in the clinical environment.

Air-borne transmission is another proposed route of transmission, for example bed making activities may release microorganisms and allow them to settle in the environment (Handorean et al., 2015). Handorean et al. (2015) studied microbial aerosol generation from the movement of soiled textiles in a newly commissioned hospital in the USA using fluorescence and molecular phylogenetic analysis. The number of microbial aerosols detected in temporary soiled textile holding rooms fitted with HEPA filters rapidly and significantly increased after the hospital was opened. The majority of bacterial isolates belonging to microbes associated with human, skin, hair and faeces: *Staphylococcus, Propionibacteria, Corynebacteria, Lactobacillus* and *Streptococcus.* The microbial aerosols detected in the terminal textile storage room also became indistinguishable from the temporary holding rooms indicating that the movement of soiled textiles between rooms generated aerosols, which may contaminate the healthcare environment. This study did not demonstrate contamination of the near-patient area from these linens, which could pose a risk of transmission to patients, or determine any link with the aerosolised microorganisms and HCAIs in the hospital.

The number of airborne MRSA increased 25-fold during bed making in hospital rooms of MRSA infected patients to 116 ± 43.7 CFU/m^3^ before returning to baseline levels by 30 min post bedmaking (Shiomori et al., 2002). MRSA was also detected on surfaces in the near-patient area 1 h after bedmaking, indicating that bedmaking may contribute to environmental contamination with healthcare associated pathogens (Shiomori et al., 2002). Although the studies of Handorean et al. (2015) and Shiomori et al. (2002) demonstrate that potential pathogens can be aerosolised and transferred to the environment by the movement of contaminated linens, these studies do not provide any evidence that HCAIs were acquired from the subsequent environmental contamination. There is currently insufficient evidence to conclude either way if the movement of textiles is a source of HCAIs or not.

Despite published studies providing a theoretical basis for healthcare linen as potential fomites, there is a lack of direct evidence linking textile contamination and the transmission of HCAIs. Interventional studies that investigate antimicrobial linen treatments on the rates of HCAIs may provide some indirect evidence for the role of healthcare textiles as fomites. A number of such trials have been conducted on copper oxide impregnated textiles. An uncontrolled pre and post intervention study was employed to determine the effect of antimicrobial copper oxide impregnated healthcare linens on the rate of HCAIs in a 35-bed head injury ward. During a 6-month period using copper oxide treated linens there was a 24% reduction in HCAIs and fevers (*p* ≤ 0.05) compared to 6 months using untreated linens. In particular there were significant reductions in gastrointestinal and eye infections. There was a significant reduction in the load of microorganisms on linens after 6–7 h patient use compared to untreated linens (46–50%). A disadvantage of this study is the small sample size (*n* = 57, control period; *n* = 51, intervention period) and the uncontrolled nature of the study design (Lazary et al., 2014). Randomised controlled trials conducted using a larger number of participants and over a longer study period would be needed to provide stronger evidence of a reduction in HCAIs associated with antimicrobial linens.

A double-blind controlled crossover study was conducted using copper impregnated textiles over two 3-month periods in chronic ventilator-dependent patients. The findings indicated that there was a significant reduction in fever days (55.5%), antibiotic treatment initiation events (29.5%) and antibiotic daily defined doses during the use of copper impregnated textiles, however microbiological data was not provided. Stratifying these HCAI indicators over time suggested that there was a significant decrease in all indicators except daily defined dose between the two study periods (Marcus et al., 2017). This indicates that HCAIs were reduced over time independently of the intervention, which confounds the effect of copper impregnated textiles against HCAIs alone. The studies of Marcus et al. (2017) and Lazary et al. (2014) were conducted on specific groups of patients (long term ventilator-dependent and head injury patients, respectively) which may influence susceptibility to HCAIs and interventions; the investigation of a wider range of patients would minimise confounding variables associated with differing participant populations. (Butler, 2018) investigated the use of copper oxide impregnated linens over 240 days in six hospitals (total 1019 beds) using an uncontrolled pre and post intervention study. There was a significant (*p* ≤ 0.05; 42.9%) reduction in *C. difficile* infections, a non-significant (*p* > 0.05; 19.2%) reduction in infections associated with multidrug resistant microorganisms and a significant (*p* ≤ 0.05; 37.2%) reduction in combined *C. difficile* and multidrug resistant infections after 240 days employing copper treated textiles. This study is advantageous in that it was conducted against a large number of patients (six hospitals; 1019 beds), yet it was noted that infection control education was implemented at the same time as the intervention, which is a confounding variable to the use of copper impregnated textiles. Moreover, the uncontrolled nature of the study design means that limited conclusions can be drawn on the efficacy of the intervention on HCAI rates. Overall, the evidence available from intervention studies on the impact of antimicrobial textiles on the rate of HCAIs is inconclusive. There is little evidence from the intervention studies to support or dispel the hypothesis that healthcare textiles are a source of HCAIs; further robust studies are needed.

**Table 3:** Case studies of healthcare-associated infection outbreaks associated with contaminated textiles.

**Microorganism**	**Outbreak**	**Concluded source**	**Reference**
*B. cereus*	Meningitis following neurosurgery (*n* = 2).	Surgical scrubs contaminated with spores.	Barrie et al. (1992)
*G. bronchialis*	Sternal infection in postoperative patients (*n* = 3).	Surgical scrubs contaminated by a domestic washer-extractor machine colonised with *G. bronchialis.*	Wright et al. (2012)
MRSA	MRSA infections across three wards (*n* = 25).	Transmission from healthcare worker attire to patients or *vice versa.*	Osawa et al. (2003)
ESBL *K. oxytoca*	Colonisation of paediatric ward patients (*n* = 14).	Knitted clothing laundered in a domestic washer-extractor machine colonised with *K. oxytoca.*	Schmithausen et al. (2019)
ESBL *K. pneumoniae*	Colonisation of rehabilitation centre patients (*n* = 14).	Contamination of clothing and lifting slings from a colonised domestic washer-extractor machine; inadequate laundering parameters for soiled laundry (30–40 °C and detergent without activated oxygen bleach).	Boonstra et al. (2020).
*Acinetobacter* sp.	Colonisation and/or infection of patients in a German hospital (*n* = 187).	High-level contamination of pillows, which was resolved by switching the laundering cycles of the pillows from 60 °C to 85 °C.	Weernink et al. (1995)
*A. baumannii*	Carbapenem-resistant *A. baumannii* infection outbreak in an intensive care unit (*n* = 13).	Privacy curtains, bed surfaces, equipment and mop heads colonised with *A. baumannii.*	Das et al. (2002)
*C. difficile*	Healthcare associated *C. difficile* infection in one hospital (*n* = 14).	Washer-extractor machine programming error where bleach was not dispensed, leading to inadequate decontamination of mop heads.	Sooklal, Khan & Kannangara (2014)
*B. cereus*	*B. cereus* bacteraemia (*n* = 11).	Contamination of linen by a continuous tunnel washer employing recycled water.	Sasahara et al. (2011)
	Colonisation of neonates with *B. cereus* in one UK hospital during the summer months (*n* = 42).	Proliferation of *B. cereus* on used linen and inadequate decontamination during laundering with a continuous tunnel washer.	Hosein et al. (2013)
	*B. cereus* outbreak in a Singapore hospital (*n* = 171).	Proliferation of *B. cereus* on linen stored in air-tight plastic bags, and high levels of *B. cereus* in air samples.	Balm et al. (2012)
*Rhizopius* sp.	*Rhizopius* sp. outbreak in hospital patients (*n* = 5).	Linen contaminated post-laundering.	Duffy et al. (2014)
	*Rhizopius* sp. outbreak in hospital patients (*n* = 4).	Linen contaminated post-laundering by linen carts which were not cleaned routinely.	Teal et al. (2016)
	Invasive cutaneous *Rhizopius* sp. infection (*n* = 6).	Linen contaminated post-laundering.	Cheng et al. (2016)

Case studies have reported small outbreaks or colonisation of patients associated with contaminated linen or laundering equipment (Sehulster, 2015). Three studies in the published literature implicate contaminated healthcare worker uniforms with HCAI outbreaks (Table 3). Barrie et al. (1992) investigated two cases of *Bacillus cereus* meningitis infections following neurosurgery in the same hospital. Environmental sampling demonstrated that surgical scrubs were contaminated with *B. cereus,* suggesting the spores were shed from the textile on to the patient (Barrie et al., 1992). A second study concluded that the source of three sternal *Gordonia bronchialis* infections in postoperative patients was the contaminated scrubs of a nurse anaesthetist; the scrubs were domestically laundered using a machine contaminated with *G. bronchialis.* The outbreak ceased upon discontinuing use of the contaminated washing machine (Wright et al., 2012). Environmental surveillance during an MRSA outbreak demonstrated that MRSA clinical isolates were related to those detected on fingers, white coats and nares of doctors and nurses using pulse field gel electrophoresis (PFGE) typing (Osawa et al., 2003). This suggests that cross-contamination of MRSA between patients and healthcare worker uniforms occurred, but it cannot be concluded if the MRSA was transmitted to patients from the healthcare worker attire or *vice versa.*

A number of other HCAI outbreaks have been attributed to the contamination of hospital linens and patient attire by washing machines (Table 3). Environmental microorganisms can colonise washing machines, such as in the drawers and door seals, and subsequently shed on to the textiles during laundering (Babic et al., 2015; Callewaert et al., 2015). This is particularly common in domestic washing machines, especially when used at low temperatures; one study reported that 79% of domestic washing machines used at 40 °C were positive for fungi (Babic et al., 2015). Environmental microorganisms may cause opportunistic infections in immunocompromised individuals which may be a risk in healthcare settings. Recently, colonisation of 13 neonates and 1 child in a paediatric hospital ward with extended spectrum β-lactamase (ESBL)- producing *Klebsiella oxytoca* was attributed to clothing laundered in a domestic washer-extractor machine on the ward. *K. oxytoca* was recovered from the detergent drawer and rubber door seal of the washing machine which was hypothesised to act as a reservoir for the contamination of textiles laundered in the machine. No further cases were reported once the machine was taken out of use (Schmithausen et al., 2019). Similarly, 14 rehabilitation centre patients were colonised with ESBL-producing *Klebsiella pneumoniae* during October–November 2016. The outbreak was again associated with a domestic washing machine used to launder soiled patient clothing and lifting slings at 30−40 °C using detergent without activated oxygen bleach. The washing machine was colonised with the ESBL-producing *K. pneumoniae*; the outbreak ceased when the washing machine was taken out of use (Boonstra et al., 2020). Despite the outbreaks reported by Boonstra et al. (2020) and Schmithausen et al. (2019) only demonstrating colonisation rather than infection, these studies provide evidence for microorganisms transmitting from contaminated textiles to patients. There have been a number of similar outbreaks, including a cluster of *Streptococcus pyogenes* infections in neonates that was linked to the contamination of vests laundered in-house; environmental sampling demonstrated that the tumble drier was contaminated with *S. pyogenes* (Brunton, 1995)*.*

HCAI outbreaks have also been associated with the use of inappropriate laundering parameters (Table 3). Feather pillows were suspected to be a fomite in an *Acinetobacter* sp. outbreak in a German hospital due to high level contamination of pillows with identical biotypes to clinical isolates. Switching the laundering cycles of the pillows from 60 °C to 85 °C eliminated *Acinetobacter* sp. contamination and subsequently the number of infection cases was reduced (Weernink et al., 1995). An outbreak of *C. difficile* infections in a hospital was associated with inadequate washing of mop heads, due to the washing machine failing to dispense bleach into the wash (Sooklal, Khan & Kannangara, 2014). Privacy curtains in an intensive care unit were reportedly colonised with Carbapenem-resistant *Acinetobacter baumannii,* leading to an outbreak of 13 infections. Environmental sampling identified *A. baumannii* isolates on bed surfaces, equipment, mop heads and privacy curtains that were indistinguishable from the clinical isolates by PFGE. The outbreak was reportedly resolved by implementing frequent changing of the privacy curtains (Das et al., 2002). The use of recycled water in a continuous tunnel washer processing hospital linen was implicated in an outbreak of *B. cereus* bacteraemia in a hospital (11 cases) during 2006. All towels and linens sampled in the laundry were positive for *B. cereus* (6.6 ×10^2^ –7.1 × 10 ^4^CFU/cm^2^) prior to intervention. The environmental *B. cereus* isolates were heterogenous, as determined by PFGE, yet there was some evidence of shared isolates between environmental and clinical samples. *B. cereus* forms spores which are resistant to alcohol; it was hypothesised that *B. cereus* was transmitted to patients during catheterisation by nurses that used alcohol gel to sanitise their hands. The load of *B. cereus* on linens was reduced to 81 CFU/cm^2^ following disinfection of the continuous tunnel washer and stopping the use of recycled water. Glove wearing during intravenous infusion procedures was implemented at the same time, which prevents conclusions being drawn about the role of reducing linen contamination alone on HCAI transmission (Sasahara et al., 2011).

The inadvertent contamination of healthcare linens after laundering has been another reported source of textile associated outbreaks (Table 3). This includes inappropriate storage conditions within the hospital or laundry that lead to contamination with potential pathogens or encourage microbial proliferation. For example, Hosein et al. (2013) reported that 65% of neonatal umbilical swabs in one UK hospital were positive for *B. cereus* in the summer of 2009. Environmental samples were negative for *B. cereus* with the exception of linen. The number of positive umbilical and linen cultures decreased to zero during the autumn and winter. *B. cereus* may replicate and sporulate on damp and soiled linen at higher temperatures during the summer months leading to greater contamination compared to the colder winter months. *B. cereus* spores are resistant to thermal disinfection during laundering; it was also hypothesised that the increased bioburden was not eluted from the linen due to the use of low volumes of water in the laundering processes to improve water-efficiency, leading to *B. cereus* spores surviving on the processed linen (Hosein et al., 2013). Hosein et al. (2013) did not further investigate the industrial laundry and the source of the linen contamination was not concluded. Proliferation of *B. cereus* on linens stored in air-tight plastic bags was reported to encourage the growth of *B. cereus* (4437 CFU/cm^2^) compared to canvas bags (166 CFU/cm^2^) and was in part attributed to an outbreak of *B. cereus* (171 cases) in a Singapore hospital. There were also high levels of *B. cereus* in air samples within the hospital, indicating that the role of healthcare textiles alone in the outbreak is not defined (Balm et al., 2012). A number of studies have attributed the contamination of linen post-laundering to outbreaks of the fungi *Rhizopus* sp.; one study reported that 42% of laundered linen samples, 45% of linen bins, 100% of linen delivery trucks and 78% of storage rooms were contaminated with *Rhizopus* sp. during an outbreak affecting five people in a hospital. The isolates detected did not correlate with the molecular type of the clinical isolates providing limited evidence of the transmission from linen to patient. Moreover, there was no definitive reservoir of *Rhizopus* identified in this study, meaning no conclusions can be drawn to at which stage of the laundering process the linens were contaminated (Duffy et al., 2014). A further *Rhizopus* sp. outbreak (four cases) was linked to contaminated linen carts used to transport laundered textiles. The industrial laundering only cleaned the carts after visible soiling and 2 out of 3 were positive for *Rhizopus* sp. There were no new *Rhizopus* infections reported after routine cleaning of linen carts (Teal et al., 2016). Widespread *Rhizopus* sp. contamination of an industrial laundry was also associated with invasive cutaneous *Rhizopus* sp. infection in six immunosuppressed patients. It was reported that 61% of environmental samples and 28% of laundered clothing items testing positive and a correlation between environmental and clinical isolates was established by phylogenetic analysis (Cheng et al., 2016).

The case studies reported in the published literature provide preliminary evidence of a link between HCAIs and textiles; their small sample size and retrospective nature makes it difficult to conclude a direct link between the contaminated linen and outbreaks. However, the outbreak case studies demonstrates that textiles are able to act as fomites and this evidence should not be ignored. Most of the outbreaks reported in the published literature were associated with opportunistic environmental bacteria rather than healthcare associated pathogens, which does not provide evidence of patient-to-patient transfer of HCAIs (Sasahara et al., 2011; Wright et al., 2012; Hosein et al., 2013; Duffy et al., 2014 Sehulster, 2015, Cheng *et al.*, 2016; Teal et al., 2016), yet the transmission of environmental microorganisms is still of importance and should be controlled due to a proportion of hospitalised patients being immunocompromised or possessing underlying co-morbidities. Large epidemiological or intervention studies are required to provide more robust evidence of any direct link between contaminated textiles and HCAIs to conclude the scale of any potential transmission through this route (Bloomfield et al., 2015).

The outbreak case studies indicate that minimising the contamination of textiles with microorganisms could reduce the risk of infections associated with healthcare textiles. This demonstrates that laundering is a critical process in ensuring the safety of healthcare textiles and that adequate decontamination should be ensured. Controls may include; ensuring adequate decontamination of linen during laundering, monitoring for contamination of washing machines and rinse water and appropriate handling and storage of processed linen to prevent contamination.

### Efficacy of healthcare laundry processes

#### Current healthcare laundry policies

The main objectives of healthcare laundering are to remove visible soiling and reduce the microbiological load, to minimise the transmission of infections and prevent malodour (Bockmühl, Schages & Rehberg, 2019). Healthcare linens are laundered industrially, employing high temperatures (≥60  °C) and/or detergents and disinfectants for cleaning and decontamination of the textiles (Bockmühl, Schages & Rehberg, 2019). Healthcare uniforms are generally washed industrially, however in some countries they are laundered domestically by the healthcare worker, for example in the UK (NHS, 2020), Republic of Ireland (Health Protection Surveillance Centre, 2020) and in some hospitals in the USA (Nordstrom, Reynolds & Gerba, 2012). General infection control measures in place in industrial laundering include the separation of areas of the laundry for dirty and clean linens and routine cleaning of the environment and equipment, which minimises the risk of recontamination of linens post-laundering, which are not present in the home environment (TRSA, 2019). For example, in the EU and UK, the EN14065 (European Committee for Standardization (CEN , 2016; British Standards Institute, 2016) Risk Analysis and Biocontamination Control (RABC) system and HTM 01-04 require laundries to determine microbiological hazards and implement control measures to ensure decontamination and prevent recontamination of linen. Parameters for each control point are monitored and target levels are established for each parameter, which infer microbiological load of processed textiles. Action levels indicate that controls have not been implemented and corrective action is required to re-establish control and prevent potential contamination of textiles. Control measures include, for example, cleaning and disinfection of laundry surfaces and equipment, functional separation of areas for handling clean and soiled linen, and laundry personnel hygiene and personal protective equipment (CEN, 2016; British Standards Institute, 2016; U.K. Department of Health, 2016a).

The UK Health and Social Care Act 2008: Code of Practice on the prevention and control of infections and related guidance (U.K. Department of Health, 2008) necessitates infection control and prevention to be incorporated into everyday processes and be applied consistently by everyone. Linens are industrially laundered, with the exception of healthcare worker uniforms which are laundered domestically (Riley, Laird & Williams, 2015). The UK Department of Health (U.K. Department of Health, 2010) and National Health Service (NHS, 2020) healthcare worker uniform policies recommend washing uniforms at the hottest temperature that the fabric can withstand and state that washing at 60 °C for 10 min removes “almost all microorganisms” (NHS , 2020) whilst washing with detergent at 30° C eliminates MRSA and most other Gram-positive bacteria, based on the results of an unpublished study (U.K. Department of Health, 2010; NHS, 2020). The U.K. Department of Health (2016a) Healthcare Technical Memorandum 01-04 (HTM 01-04) forms essential and best practice guidance for industrial laundering healthcare linen to maintain the safety of patients. Infected linen should be enclosed in water-soluble alginate bags that are placed directly in the washer to avoid handling and pre-sorting of the linen. The HTM 01-04 recommends that disinfection of linen is achieved thermally, at 10 min at 60 °C or 3 min at 71 °C with further mixing time based on the weight of the load. Chemical disinfection may be employed at reduced temperatures if the process is equal to or more effective than thermal disinfection (Table 4).

**Table 4:** Domestic and industrial laundering parameters used for healthcare textiles according to national policies within the UK, Germany and USA.

**Country**	**Laundering parameters**	**References**
United Kingdom	*Domestic Laundering (NHS healthcare uniforms)* Hottest temperature that the fabric can withstand; policy states that washing at 60 °C for 10 min removes “almost all microorganisms” whilst washing with detergent at 30° C eliminates MRSA and most other Gram-positive bacteria. *Industrial Laundering (linen and scrubs)* Thermal Disinfection: 60 °C for 10 min or 71 °C for 3 min plus mixing time based on weight of the load. Chemical Disinfection: lower temperatures are permitted if the process is equal to or more effective than thermal disinfection.	NHS (2020) U.K. Department of Health (2016a)
Germany	*Industrial Laundering* Thermal Disinfection: 90 °C for 10 minutes Chemical Disinfection: Using an approved agent outlined by the RKI.	Fijan, Cencic & Turk (2006); Heintz & Bohnen (2011)
United States of America	*Industrial Laundering* Thermal disinfection: ≥71 °C for 25 min in a wash with detergent Chemical Disinfection: Chemicals used should be suitable for use at low temperatures	CDC (2003)

In Germany, the Robert Koch Institute (RKI, 2003) guidelines state the hygiene requirements for commercial laundries, such as separation of soiled and clean linen and appropriate loading of washing machines (Heintz & Bohnen, 2011). Thermal disinfection of 90  °C for 10 min (Fijan, Cencic & Turk, 2006), and chemical disinfection must be conducted using a given list of agents outlined by the RKI (Heintz & Bohnen, 2011; Table 4).

In the USA, the Centers for Disease Control and Prevention (CDC) states that laundry should be rendered ‘hygienically clean’, defined as generally free from vegetative pathogens but not sterile (CDC, 2003). Thermal disinfection of ≥71 °C for 25 min in a wash with detergent is recommended, and if low temperature washing (<71 °C) is used, disinfectant chemicals selected should be suitable for use under low temperatures (CDC, 2003; Table 4).

### Current healthcare laundry test methods

The HTM 01-04 states that washing parameters (e.g., temperature) must be monitored during use to ensure disinfection parameters are achieved (U.K. Department of Health, 2016b). Decontamination of microorganisms by a laundering process should be routinely validated by a swatch test based on the BS EN ISO 14698 appendix E method (British Standards Institute, 2016); a sterile textile swatch should remain sterile within the laundering process. Additional best practice validation for HTM 01-04 employs commercially available biological indicators, comprised of textile swatches inoculated with *Enterococcus faecium* and surrounded by a semi-permeable membrane (Table 5)*.* The laundering process should reduce *E. faecium* by 5 log_10_, demonstrating equivalent activity to thermal disinfection. *Enterococcus* sp. is used because it is a thermotolerant microorganism that can survive at commonly employed temperatures for decontamination of healthcare laundry. Orr et al. (2002) reported that only two out of 40 *Enterococcus* sp. isolates were reduced by greater than 5 log_10_ by exposure to 71 °C for 3 min or 10 min at 65 °C (Orr et al., 2002). The use of a semi-permeable membrane prevents dilution of microorganisms away from the test swatch to ascertain the true kill by heat and disinfectant parameters, however the method is only semi-quantitative and does not provide accurate log_10_ reduction numbers. The HTM 01-04 also specifies that *B. cereus* spore testing should be conducted monthly during June to September, yet no method and action level is prescribed by the policy, which may lead to variation in the recovery and the perceived decontamination efficacy.

**Table 5:** Currently employed methods to determine the efficacy of industrial laundering and microbiological burden of textiles.

**Country**	**Test**	**Method**	**Pass criteria**	**Reference**
United Kingdom	Sterile Swatch Test	Laundering of sterile textile swatch and viable counting number of contaminating microorganisms by eluting in recovery media and membrane filtration.	No microorganism detected.	British Standards Institute (2016), U.K. Department of Health (2016b)
	Semi-permeable dosing strips	Semi-permeable membranes containing *Enterococcus faecium* on textile carriers are laundered. Textile swatches are removed from the semi-permeable membrane and incubated in tryptone soya broth. A lack of growth indicates a 5 log_10_ reduction.	5 log_10_ reduction of *E. faecium.*	U.K. Department of Health (2016b).
Germany (RKI/RAL)	Bioindicator method	Cotton carriers inoculated with *S. aureus* or *E. faecium* are laundered before incubation in tryptone soya broth. A lack of growth indicates a 5 log_10_ reduction.	5 log_10_ reduction of *E. faecium* and *S. aureus.*	Fijan, Cencic & Turk (2006), Heintz & Bohnen (2011).
	RODAC plating	RODAC plates are pressed on to the surface of a defined area of laundered textile. After incubation, the number of colonies are counted. Colonies are further investigated for specific pathogens.	≤20 CFU/dm^2^ microorganisms. RAL: no pathogens; ≤30 CFU/dm^2^ on damp textile, <100 CFU/dm^2^ on environmental surfaces.	Fijan, Šostar Turk & Cencič (2005), Heintz & Bohnen (2011).
United States of America (TRSA)	USP 62	Processed textile is incubated in tryptone soya broth and plated onto selective agars for *Salmonella* sp., *P. aeruginosa, S. aureus, Clostridium* sp. and *C. albicans.*	No pathogens detected.	United States Pharmacopoeia (2016); TRSA (2019).
	RODAC Plating	RODAC plates are pressed on to the surface of a defined area of laundered textile. After incubation, the number of colonies are counted.	≤20 CFU/dm^2^ microorganisms; ≤20 CFU/dm^2^ yeasts and mould total count.	

In Germany, laundering processes are monitored by sampling of processed linen using RODAC contact agar plates (Table 5). The RKI also uses a swatch testing method to determine the efficacy of washing processes where samples of cotton inoculated with *E. faecium* or *S. aureus* are laundered. No growth should be observed, indicating a 5 log_10_ reduction (Fijan, Cencic & Turk, 2006; Heintz & Bohnen, 2011). Similar to the UK, after validation of the process the washing parameters are then monitored during routine use to ensure disinfection has been achieved (Fijan, Cencic & Turk, 2006). The German Institute for Quality Assurance and Certification (RAL) implements the RAL-GZ 992/2 standard for quality assurance of healthcare textiles based on RKI methodology, which has been adopted by commercial laundries in several European Union countries. Microbiological levels at critical control points in laundering facilities are outlined in RAL-GZ 992/2 in addition to those outlined above for the RKI, including <30 CFU/dm^2^ on damp textiles and <100 CFU/dm^2^ on environmental surfaces including technical equipment, storage shelves and laundry worker hands (Fijan, Šostar Turk & Cencič, 2005).

Contrary to UK, Germany and EU recommendations, the CDC states that routine microbiological testing of processed textiles is not required; such testing should only be performed during outbreak investigations to determine any epidemiological involvement of the healthcare textiles (CDC, 2003). The USA Textile Rental Services Association (TRSA) offers ‘Hygienically Clean Healthcare’ certification as a quality assurance standard for producing reusable healthcare textiles. The certification is based on compliance with microbiological testing, facility inspections and best management practice documentation e.g., of linen processing procedures and equipment maintenance. Microbiological testing is performed before certification and then on a quarterly basis, where two processed textile items (one flat and one terry item) are tested on a rotating basis (TRSA, 2019). The microbiological burden of the processed textiles are determined by RODAC surface tests and the United States Pharmacopoeia (United States Pharmacopoeia, 2016) 62 method (TRSA, 2019) which detects the presence of selected pathogens in processed linen (Table 5).

Despite recommendations given in the UK and German national policies to validate laundering processes for the decontamination of healthcare textiles and monitor the bioburden of processed linens, there is no standardised test methodology to ensure that the sensitivity and reliability of methods being employed are consistent across the sector. For example, agitation methods to recover microorganisms from swatch tests outlined in the U.K. Department of Health (2016b) HTM 01-04 and BS EN 14698 appendix E are not defined. Previous research has shown variation in recovery of microorganisms based on agitation method; Tarrant, Jenkins & Laird (2018) reported that vortexing 100% cotton swatches for five ×  25 s recovered significantly (*p* ≤ 0.05) greater numbers of *C. difficile* spores than by stomaching (4.41 log_10_ versus 4.11 log_10_ CFU/mL, respectively), indicating that laundry operators following the HTM 01-04 may be employing different methods. Moreover, the use of surface testing of processed linen (e.g., RODAC plating) such as in the TRSA and RKI guidelines may recover fewer microorganisms than elution methods, where samples are agitated in recovery media, due to limited contact with microorganisms trapped in the weave of textiles. This was concluded by Rabuza, Šostar Turk & Fijan (2012), where the recovery of *S. aureus* and *K. pneumoniae* from textile samples was approximately 2 log_10_ lower from RODAC plating than from shaking in recovery media for 10 min at 300 rpm. There are also clear deviations in the threshold (action) levels of microorganisms to demonstrate that decontamination has been achieved between countries (Table 5), indicating that different standards of decontamination are required between laundry operators. Standardisation of microbiological methods and action levels employed in healthcare laundries would further strengthen the current validation of wash processes and bioburden of processed textiles is consistent across the sector and meets the required standard of decontamination to ensure the infection risk from processed laundry is low.

### Efficacy of industrial laundering

The overall cleaning performance of a laundry cycle is determined by the temperature, chemistry (detergent/disinfectant), mechanical action and contact time (Bloomfield et al., 2015; Bockmuhl, 2017). These parameters are correlated in a manner where the reduction in cleaning performance from reducing one variable can be offset by increasing another variable; for example, reducing the temperature can be compensated by increasing the length of the cycle (Bockmuhl, 2017). A typical industrial laundry cycle includes detergent to remove gross soiling from the textile (CDC, 2003). Detergents are not typically antimicrobial however they lift microorganisms from textiles (CDC, 2003; Riley et al., 2017; Bockmuhl, 2017). Dilution in water also reduces the microbiological load. Temperature and/or bleaching agents such as sodium hypochlorite, activated oxygen bleach or peracetic acid disinfect the linen. Finally, a sour rinse is used to neutralise alkalinity from detergent and chlorine bleaching agents (CDC, 2003).

The outbreak case studies highlight contamination of textiles during laundering or inadequate decontamination during laundering as a potential route of patient-to-patient transmission. It is currently assumed that the risk of infection from linens laundered commercially under current guidelines is low (Loveday et al., 2007), yet a few studies have reported the survival of potentially pathogenic microorganisms on textiles during industrial laundering. Microbiological examination of laundered nursing home staff gowns showed a median (*n* = 58) of 2 CFU/25 cm^2^ Gram-positive cocci, while *S. aureus* and *E. coli* were each isolated from one of 58 laundered gowns sampled. Although the load of Gram-positive cocci recovered was lower than used, unlaundered gowns (80 CFU/25 cm^2^), this data indicates that some pathogens may survive industrial laundering processes or cross-contaminate clean laundry post washing (Heudorf et al., 2017); the infection control risk of the low microbiological load is unknown. Fijan et al. (2007) reported that 1.5 ×10^2^ –3.98 ×10^3^ CFU *Enterobacter aerogenes, S. aureus* and *P. aeruginosa* survived on textiles with artificial soiling during industrial laundering with detergent and bleaching agent at 60 °C; *E. faecium* survived to a greater extent at 60 °C (5.74 ×10^4^−5.73 ×10^6^ CFU, dependent on soiling used). The study investigated the survival of microorganisms as the wash cycle reached 60 °C rather than allowing the temperature to be held for a period of time to allow disinfection; laundering standard policies of the UK (60 °C, 10 min; U.K. Department of Health, 2016b), USA (71 °C for 25 min; CDC, 2003) or Germany (90 °C for 10 min; Fijan, Cencic & Turk, 2006) were not followed (Fijan et al., 2007). No microorganisms survived washing at 75 °C for 9 min, where the time and temperature used meets current industrial laundering policies of the UK (U.K. Department of Health, 2016b) but not the USA (CDC, 2003) and Germany (Fijan, Cencic & Turk, 2006). Laundering the uniforms of wastewater treatment plant workers in accordance with ASTM F1449 industrial laundering standards (60 °C for 25 min with detergent) reduced heterotrophic plate counts by ≤0.25 log_10_ CFU, leaving 4.1 ×10^4^ −7.5 ×10^7^ CFU remaining; 7.6 ×10^3^−4.9 ×10^4^ CFU *P. aeruginosa* was also recovered from uniforms after laundering. *E. coli, S. aureus,* MRSA, *C. difficile* and *Acinetobacter* spp. were significantly reduced by industrial laundering (0–2 ×10^2^ CFU remaining). Tumble drying further reduced the microbiological load, with 0–6 CFU total heterotrophic microorganisms and zero pathogenic microorganisms recovered (Maal-Bared, 2019). Laundering at 60 °C for 25 min meets UK DH recommendations (U.K. Department of Health, 2016b), however does not meet the UK standard of 71 °C for 3 min (U.K. Department of Health, 2016b) or USA and German standards (71 °C for 25 min or 90  °C for 10 min; CDC, 2003; Fijan, Cencic & Turk, 2006).

*C. difficile* spores are of particular concern due to their thermotolerance and resistance to disinfection and they have been shown to survive industrial laundering in line with current policies. Cotton bed sheets artificially contaminated with 7 log_10_ CFU/25 cm^2^ *C. difficile* spores retained 4.95–5.27 log_10_ CFU/25 cm ^2^ during a simulated industrial washer extractor cycle using thermal disinfection parameters (≥71 °C for >3 min). A further 2.72–2.89 log_10_ CFU/ 25 cm^2^ cross contaminated sterile textiles in the same wash indicating that the spores were not killed, but rather removed from the inoculated swatches and deposited on to other textiles. Washing at elevated temperate alone did not meet requirements of a >5 log_10_ reduction for sporicidal activity. A thermal wash with industrial bleach detergent was more effective with recovery of only 0–9 CFU/25 cm^2^ of the 7 log_10_ CFU/cm^2^ inoculum and cross contamination was limited to 0–8 CFU/ 25 cm^2^*. C. difficile* spores also survived on naturally contaminated bed linen in an industrial laundry, where the sheets were washed with industrial detergent, dried and pressed (175 °C, 4 bars pressure, 3 s). The process only resulted in a 40% reduction in viable spores recovered from the linen, from 51 CFU/25 cm^2^ to 33 CFU/25 cm^2^, demonstrating the potential survival of *C. difficile* spores under realistic conditions (Tarrant, Jenkins & Laird, 2018).

Cross contamination of sterile textiles with *C. difficile* spores was also observed in a typical healthcare linen wash cycle employing detergent, chlorine (50 ppm), peroxide (100 ppm) and peracid (54 ppm) as bleaching agents. The number of spores surviving on the inoculated swatches or those cross contaminating sterile textiles were not enumerated, rather growth was detected by incubating textiles in Brain Heart Infusion broth (Hellickson & Owens, 2007); enumeration would provide further evidence of the possible infection control risk posed by the *C. difficile* spores in relation to the infectious dose. *C. difficile* spores were also found to survive to a lesser extent in a simulated continuous tunnel wash process.

Continuous tunnel washers have been increasingly adopted over traditional washer extractor machines for industrial laundering in some countries; they are more efficient in terms of thermal energy and water consumption. Continuous tunnel washers feature a number of connecting compartments with different wash liquors that the textiles move through at a constant rate, compared to the single compartment of washer extractor machines. Washing with a sequence of alkali (45 °C, 3 min), alkali and detergent (73 °C, 6 min), peracetic acid (73 °C, 6 min), water (45 °C, 6 min) and an acid sour (45 °C, 3 min) reduced *C. difficile* spores by >3.08 log_10_ CFU/ml, compared to a 1.64 log_10_ CFU/ml reduction when washed with water alone. The greatest reduction occurred following peracetic acid treatment; 3.68 log_10_ CFU/ml survived the alkali and detergent stages prior to peracetic acid treatment, where less than 1.3 log_10_ CFU/ml survived ( McLaren et al., 2019). The study was conducted in a laboratory-scale continuous washing machine model, thus further research is needed to determine the efficacy under realistic conditions.

The varying washing processes and experimental methodologies makes comparisons between studies difficult and further research is needed to determine the extent to which *C. difficile* spores survive under different wash processes employed throughout the sector. The aforementioned studies indicate that industrial laundering processes may not sufficiently remove *C. difficile* spores from contaminated bed sheets, which could transfer to non-contaminated sheets during the wash cycle and act as a source of *C. difficile* outbreaks, in a similar manner to that described for *B. cereus* (Barrie et al., 1992; Sasahara et al., 2011; Balm et al., 2012). There is a lack of epidemiological evidence in the published literature that links *C. difficile* infection and inadequate decontamination of linens, which would be required to ascertain any such risk, aside from one outbreak study associated with mop head contamination (Sooklal, Khan & Kannangara, 2014).

There is limited published research on the survival of viruses within industrial laundry processes and little is known about the risk of viral transmission by processed linens. The differing environmental stability, disinfectant susceptibility and transmission dynamics between viruses and bacteria means that this cannot be inferred from bacterial studies. Fijan, Cencic & Turk (2006) detected rotavirus RNA on infected linen and towels after laundering in a continuous tunnel washer under RKI thermal (80 °C, 15 min) and chemo-thermal disinfection cycles. The detection of RNA alone does not infer the presence of infectious virus; therefore, this study does not demonstrate whether the rotavirus survived the laundering process and would pose a risk of infection. There do not appear to be any studies in the published literature on the survival of coronaviruses within laundering processes, which would be required to evaluate any risk of SARS-CoV-2 transmission from laundry during the COVID-19 pandemic.

There are few studies in relation to the survival of microorganisms through industrial laundering processes. It has been demonstrated that microorganisms can survive a range of industrial laundering processes, however the majority of studies appear to be conducted at lower temperatures or using bacteria spores which are significantly more resistant to disinfection than vegetative cells. The significance of the contamination observed on textiles following industrial laundering in relation to the transmission of HCAIs is not well understood; with only a small number of outbreak case studies investigating epidemiological link, the evidence of textiles laundered in line with industrial guidelines posing a major infection control risk is inconclusive. Routine microbiological monitoring of the wash cycle for antimicrobial efficacy and processed linen bioburden ensures any such risk is minimised. The survival of microorganisms through industrial laundering suggests that they would be more likely survive domestic washing, where wash processes employed are not validated or monitored, and are often conducted at lower temperatures for shorter periods of time (Honisch, Stamminger & Bockmühl, 2014; Riley, Laird & Williams, 2015).

### Efficacy of domestic laundering

In the UK and in some hospitals in the USA, healthcare worker uniforms are laundered domestically. The UK U.K. Department of Health (2010) and NHS (2020) policies suggest that healthcare worker uniforms do not pose an infection risk, citing two 2007 literature reviews which found a lack of evidence to suggest a link between contaminated healthcare worker uniforms and HCAIs. It is stated that washing at 60 °C for 10 min removes most microorganisms whilst washing with detergent at 30 °C eliminates MRSA and most other Gram-positive bacteria, however, the data underpinning these recommendations has not been published. In contrast, more recent studies demonstrate the survival of microorganisms laundered at low temperatures. In a 40 °C domestic wash cycle with biological detergent, 3.08–3.81 log_10_ CFU *E. coli* and 3.42–3.38 log_10_ CFU *S. aureus* survived on inoculated polycotton and polyester swatches, and 3.05–3.46 log_10_ CFU *E. coli* and *S. aureus* transferred on to other textiles in the wash. Washing at 60 °C completely reduced the microorganisms in accordance with (U.K. Department of Health, 2010) and (NHS, 2020) recommendations (Riley et al., 2017). A significant proportion (44%) of nurses wash their uniforms at temperatures lower than 60 °C, with 33% using 40  °C. Nurses also frequently laundered their uniforms with domestic clothing (40%), in contrast to guidance to wash uniforms separately (Riley, Laird & Williams, 2015). Taken together, the studies of Riley, Laird & Williams (2015) and Riley et al. (2017) indicate that domestic laundering could be a potential route of cross contamination to other textile in the wash and provide a potential route for microorganisms to re-enter the clinical environment. Similarly, 3–5 log_10_ CFU MRSA and 4–5 log_10_ CFU *A. baumannii* survived in a 40 °C wash cycle (10–20 min) without detergent, while complete inhibition (>7 log_10_ CFU reduction) was achieved in a wash cycle at 60 °C for 10 min without detergent (Lakdawala et al., 2011). The use of detergent (biological and non-biological) in a 30 °C wash completely reduced MRSA (>7 log_10_CFU) in support of U.K. Department of Health (2010) and NHS (2020) policy, and in contrast to the findings of Riley et al. (2017), however 3–5 log_10_ CFU *A. baumannii* survived at 30−40 °C; washing at 60 °C was still required to completely reduce *A. baumannii* (>7 log_10_ CFU) when detergent was used (Lakdawala et al., 2011). Patel, Murray-Leonard & Wilson (2006) also demonstrated that domestic laundering of scrub uniform samples at 40 °C or 60 °C completely removed *S. aureus.* High levels of Gram-negative bacteria contaminated the textile samples after washing at 40−60 °C (>1 ×10^10^ CFU/ml). The load of Gram-negative bacteria was reduced to 1.9 ×10^9^ CFU/ml at 40 °C and 4.7 ×10^3^ CFU/ml at 60 °C by air drying the textile and further decreased upon ironing (6–50 CFU/ml) or tumble drying (66–330 CFU/ml), with no microorganisms being detected following washing, tumble drying and ironing. This study demonstrates that contamination of textiles may occur during the washing process, and that drying processes can inactivate contaminating microorganisms. Honisch, Stamminger & Bockmühl (2014) investigated the efficacy of domestic laundering using activated oxygen bleach (AOB) and non-AOB containing detergents. The removal of *S. aureus, Enterococcus hirae, P. aeruginosa, C. albicans* and *Trichophyton mentagrophytes* was greater using AOB detergents compared to non-AOB detergents at low temperatures, with decreasing survival being observed at higher temperatures and longer washing times. For example, 4.9–5.6 log_10_ CFU *S. aureus* survived washing with non-AOB detergent for 15 min at temperatures from 20.5–41.8 °C and 3.0 log_10_ CFU survived at 46.7 °C. *S. aureus* was completely reduced (>6.7 log_10_) at 52–57.3 °C. Conversely, 3.6 log_10_ CFU *S. aureus* survived at 20.5–27.6 ° C and a complete reduction was achieved at 37.2–57.3  °C using AOB detergent. This highlights that the efficacy of laundering is related to the washing programme and detergent used, which is not controlled in domestic settings compared to industrial laundries.

Few published studies have investigated the survival of viruses during domestic laundering, which is of particular importance during the COVID-19 pandemic to prevent any risk of cross-contamination of SARS-CoV-2 from healthcare worker uniforms. There do not appear to be any published studies that have investigated the survival of coronaviruses during laundering. Enteric viruses have been found to survive domestic laundering; 3.6–4.1 log_10_ rotavirus, hepatitis A virus and adenovirus survived in a cold (20−23 ° C) wash with domestic detergent, with the removed virus mainly being transferred on to sterile textile in the wash (2.7–3.3 log_10_). In a wash with household bleach (114-125 mg/l free chlorine in wash water) in addition to detergent, 1.8–2.6 log_10_ rotavirus, hepatitis A and adenovirus survived (Gerba & Kennedy, 2007). The effect of temperature upon inactivation of the viruses was not determined and could improve the reductions observed and it cannot be concluded as to the survival of viruses on textiles laundered at 60° C as recommended by U.K. Department of Health (2010) and NHS (2020).

A concern with domestic washing is the lack of routine microbiological testing compared to industrial laundering which could lead to undetected contamination of healthcare worker uniforms with potential pathogens. Domestic washing machine are often colonised with microorganisms which can be deposited onto textiles during laundering, posing a risk of cross contamination in the clinical environment (Patel, Murray-Leonard & Wilson, 2006; Wright et al., 2012; Babic et al., 2015; Callewaert et al., 2015; Schmithausen et al., 2019) Domestic washing machine equipment failure poses a further risk of inadequate decontamination of textiles (Sooklal, Khan & Kannangara, 2014), domestic washing machines often fail to reach the programmed temperatures (Patel, Murray-Leonard & Wilson, 2006; Bloomfield et al., 2015). There is also an increasing use of low temperature and short wash cycles to improve energy efficiency, and due to the unsuitability of some fabrics for higher wash temperatures (Honisch, Stamminger & Bockmühl, 2014; Bloomfield et al., 2015). In this manner, a lack of compliance with uniform policies may also increase the risk of contamination with potential pathogens (Riley, Laird & Williams, 2015). Another concern with domestic laundering is the potential contamination of domestic surfaces during handling of the contaminated uniforms.

It has previously been stated that there is little evidence of domestic laundering being inferior to industrial laundering for decontamination of microorganisms (U.K. Department of Health, 2010; NHS, 2020). However, Nordstrom, Reynolds & Gerba (2012) indicated that bacteria may persist on domestically laundered healthcare worker uniforms to a greater extent than industrial laundering. Significantly lower numbers of bacteria were recovered from industrially laundered scrubs (4  CFU/cm^2^) than domestically laundered scrubs (*p* ≤ 0.05; 143 CFU/cm^2^), and none of the industrially laundered scrubs sampled were positive for Gram-positive bacteria or coliforms, in contrast to domestically laundered samples where 69–79% were positive. Domestic laundering may therefore pose a greater risk of cross-contamination in the domestic and healthcare environment. Conflicting studies have reported no difference in microbiological contamination between domestic and industrial laundering of healthcare attire, with no pathogenic bacteria detected using either method (Jurkovich, 2004). Chiereghin et al. (2020) also reported that a similar number of microorganisms remained on textiles contaminated with 700 CFU/25 cm^2^ *Enterococcus faecalis, E. coli, P. aeruginosa, Klebsiella pneumoniae* and *S. aureus* following industrial laundering in a continuous tunnel washer (with drying and ironing in a tunnel drier) and domestic laundering with biological detergent at 40 °C and 90 °C (with tumble or air drying and ironing). Few surviving microorganisms were recovered from all treatments, ranging 1–9 CFU/25 cm^2^; the number of surviving microorganisms for individual treatments were not reported and therefore differences in efficacy between the wash cycles cannot be concluded from this study. A similar number of microorganisms also survived on naturally contaminated white coats (worn for 5–7 days) after industrial and domestic laundering (5–35 CFU/25 cm^2^). The microorganisms recovered were mainly environmental bacteria, but some species could cause opportunistic infections such as *Micrococcus luteus* and *Staphylococcus epidermidis* (Chiereghin et al., 2020). The published reports of Nordstrom, Reynolds & Gerba (2012), Jurkovich (2004) and Chiereghin et al. (2020) support the assertion that industrial laundering is not significantly more effective than domestic laundering. These studies were small scale investigations and it should be noted that the simulated laundering cycles may not represent those used by all healthcare workers. Further research is needed to establish any difference in laundering domestically and industrially, and the subsequent risk of microbiological transmission from inadequately decontaminated healthcare textiles (Table 6).

**Table 6:** Summary of conclusions drawn from the current published literature and knowledge gaps relating to the role of textiles as fomites in the healthcare environment.

**Subject area**	**Conclusion from current literature**	**Knowledge gaps**
Contamination of Healthcare Textiles	Potential pathogens have been shown to contaminate the near-patient environment and healthcare worker attire. In vitro studies demonstrate that microorganisms can persist on textiles for several days. Textiles could therefore act as a reservoir for microorganisms, if they are able to transfer to other surfaces in sufficient numbers to cause disease.	In vitro studies may not adequately reflect in use conditions which might affect the observed survival. In particular, the load of microorganisms employed are often higher than natural levels of contamination, simulated soiling is used infrequently, and survival after dry transfer is not measured.
The Role of Healthcare Textiles in the Transmission of Infection	Microorganisms transfer between textiles and surfaces with less efficiency than non-porous surfaces. There is preliminary evidence for the transfer of microorganisms during simulated clinical activities, bedmaking and transportation of soiled linens. Outbreak case studies provide preliminary evidence of a link between HCAIs and textiles. The outbreak case studies indicate that minimising the contamination of textiles with microorganisms could reduce the risk of infections associated with healthcare textiles. Controls may include ensuring adequate decontamination of linen during laundering, monitoring for contamination of washing machines and rinse water and appropriate handling and storage of processed linen to prevent contamination.	There is a lack of direct evidence linking textile contamination and the transmission of HCAIs. High-quality controlled trials are required to provide evidence for the transmission of potential pathogens, or lack thereof, from healthcare textiles in the clinical environment. The small sample size and retrospective nature of outbreak case studies makes it difficult to conclude a direct link between the contaminated linen and outbreaks. Large epidemiological or intervention studies are required to provide more robust evidence of any direct link between contaminated textiles and HCAIs to conclude the scale of any potential transmission through this route.
Efficacy of Healthcare Laundry Processes	There is some evidence to suggest that potentially pathogenic microorganisms survive domestic laundering, particularly where conducted at low temperatures rather than those recommended by uniform policies. A disadvantage of domestic laundering is the lack of control and monitoring for decontamination compared to industrial laundering. Outbreak case studies have provided preliminary evidence for the transmission of infection by contaminated domestic washing machines, suggesting that contaminated healthcare worker uniforms could pose a risk of transmitting potential pathogens back into the clinical environment. Microorganisms, particularly thermotolerant species or spores, can survive industrial laundering processes.	Few published studies have investigated the survival of viruses during domestic laundering, which is of particular importance during the COVID-19 pandemic to prevent any risk of cross-contamination of SARS-CoV-2 from healthcare worker uniforms. There do not appear to be any published studies that have investigated the survival of coronaviruses during laundering. The significance of the contamination on industrially laundered textiles is not well understood, with only a small number of outbreak case studies investigating epidemiological links. There is a lack of standardised test methodology across the industrial laundering sector which would ensure the infection risk from processed laundry is low across the sector.

There is some evidence to suggest that potentially pathogenic microorganisms survive domestic laundering, particularly where conducted at low temperatures rather than those recommended by uniform policies. Adequate decontamination of healthcare worker uniforms is of particular importance during the COVID-19 pandemic to reduce any potential transmission *via* this route. Critically, industrial laundering processes are routinely monitored to ensure that textiles are decontaminated, and infection control procedures are in place to minimise potential cross-contamination (such as maintenance of washing machines, routine environmental disinfection and the physical separation of areas for clean and dirty linen) which is not possible with domestic laundering. The lack of control and monitoring associated with domestic laundering, and the lack of compliance with domestic laundering policies (Riley, Laird & Williams, 2015) poses the risk of undetected inadequate decontamination and cross contamination to both the domestic and clinical environments (Riley et al., 2017). Indeed, outbreak case studies have indicated the transmission of infection by contaminated domestic washing machines (Wright et al., 2012). In house or industrial laundering of healthcare worker uniforms would mitigate this risk due to implementation of process controls and microbiologically validated wash cycles.

## Conclusions

Potentially pathogenic microorganisms can survive on textiles for extended periods of time, and evidence suggests their survival during laundering (Table 6). The role of contaminated linen in the transmission of infections is debated. Inadequate laundering or infection control surrounding laundry processes have been implicated in small outbreaks in healthcare settings, however the evidence for healthcare textiles posing a major infection control risk is currently inconclusive, warranting further study. Sufficient microbiological decontamination and regulation of laundering practices is required to minimise the risk of sporadic infectious disease outbreaks in the healthcare environment. Studies have identified outbreaks associated with contaminated domestic washing machines, indicating that the domestic laundering of healthcare worker uniforms could be a potential route for transmission of potential pathogens into the clinical environment. The lack of compliance with domestic laundering policies, difficulties in implementing infection control procedures and variation in the performance of domestic washing machines may enhance the risk of inadequate microbiological decontamination of healthcare worker uniforms compared to those that have been industrial laundered. Within industrial launderers, the use of a standard method to detect contamination of healthcare laundry with potential pathogens would ensure comparable microbiological decontamination of healthcare laundry across the sector to minimise the potential risk of cross contamination to the clinical environment.

## Additional Information and Declarations

### Data Availability

The following information was supplied regarding data availability: No data was generated as this is a literature review.
